# Adjustment of traumatic femur shortening assisted by an intramedullar skeletal kinetic distractor: a case report

**DOI:** 10.1186/1752-1947-7-217

**Published:** 2013-08-23

**Authors:** Sascha Rausch, Kajetan Klos, Florian Gras, Marco Dutschke, Gunther O Hofmann, Thomas Mückley

**Affiliations:** 1 Department of Traumatology, Hand and Reconstructive Surgery, Friedrich-Schiller-Universität Jena, Erlanger Allee 102, Jena 07740, Germany; 2 Department of Traumatology and Orthopaedic Surgery, Helios Klinikum Erfurt, Nordhäuser Straße 74, Erfurt 99089, Germany

## Abstract

**Introduction:**

Distal comminuted femoral fractures with joint involvement are highly challenging for the surgeon. We present a potential therapeutic concept that aims especially at the treatment of posttraumatic leg length discrepancy.

**Case presentation:**

This case report describes a polytraumatized 19-year-old German woman. Among other injuries she had a third grade open distal comminuted femoral fracture with a long distance metaphyseal osseous defect. As a primary care procedure an external fixation was applied at first. On day 13, an open reconstruction of her distal femur and the articular surface was performed by screw osteosynthesis, shortening and intramedullary nailing. Due to delayed osseous consolidation an autologous cancellous bone grafting was performed twice. In addition to the second cancellous bone graft an allogeneic cortical bone graft was implemented. A 6.5cm posttraumatic leg length shortening after osseous consolidation was the result. The entire leg length shortening was successfully treated 16 months after her accident with the help of an intramedullar skeletal kinetic distractor.

**Conclusions:**

With the help of the current case report of a patient with polytrauma and a third grade open distal comminuted femoral fracture with joint involvement and a long distance osseous defect, we present a potential therapeutic concept that aims especially at the treatment of posttraumatic leg length discrepancy.

## Introduction

Distal comminuted femoral fractures with joint involvement are highly challenging for the surgeon. Resulting posttraumatic discrepancy of leg length leads to an awry pelvis and the compensation mechanism causes a scoliosis. We present a potential therapeutic concept that aims especially at the treatment of posttraumatic leg length discrepancy. The use of an external fixation is the most common technique used with its capability of leg lengthening and correction in axis deviation. In this case report the limb length compensation was performed with, after ruling out any angular or rotational deformities, the implant of an intramedullar skeletal kinetic distractor (ISKD; Orthofix, Ottobrun, Germany). In the case of the right indication, and by using it correctly, this device rises well above other lengthening devices being used currently.

## Case presentation

**Figure 1:**
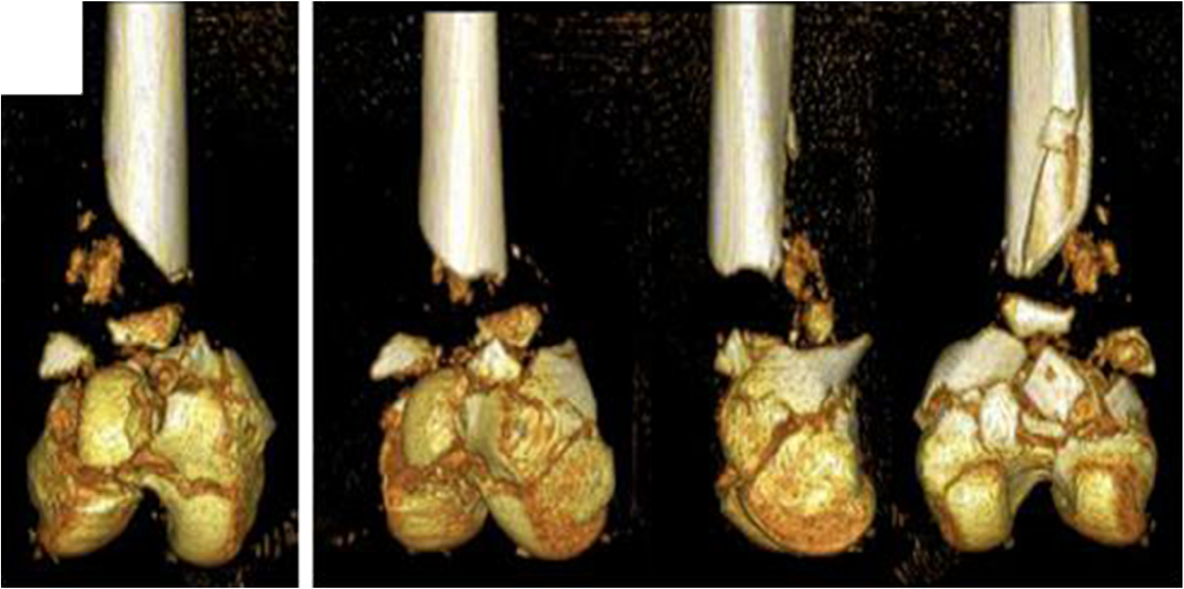
Preoperative imaging (three-dimensional reconstruction computed tomography scan).

A 19-year-old German woman with polytrauma (Injury Severity Score [ISS] 27, Pediatric Trauma Score [PTS] 29) had a third grade open distal comminuted femoral fracture with a long distance metaphyseal osseous defect (AO-Cl: 33-C3.3) and she also lost a multiple centimeter large fragment of the diaphysis in a car accident (Figure 1). Further injury was an osseous avulsion fracture of her posterior cruciate ligament, a rupture of her quadriceps tendon as well as a Lisfranc luxation of her right lower limb. The patient additionally had a second degree traumatic brain injury with multiple contusion hemorrhage and a lung contusion on the left side. The initial treatment took place a half-hour after the accident. Therefore an external fixator, including the joint of her knee, was applied as well as the suture of her quadriceps tendon and surgical wound care of the multiple contused lacerated wounds.

**Figure 2:**
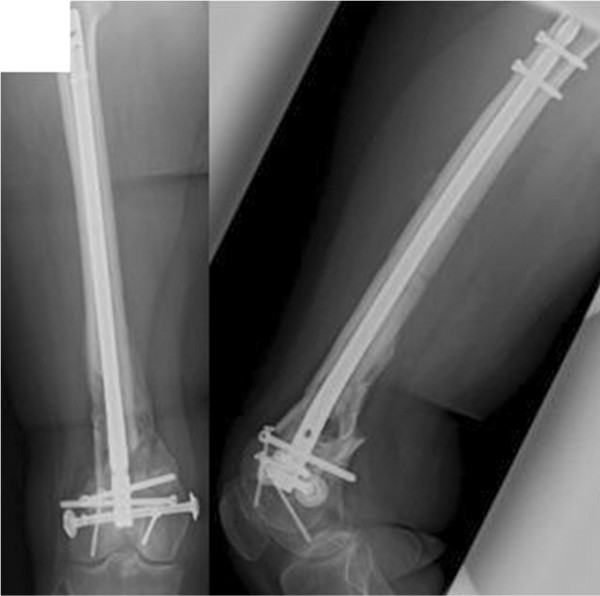
Postoperative imaging (X-ray).

After stabilizing the vital parameters and conditioning the soft tissue, the external fixator was exchanged through a retrograde intramedullary nail. The procedure started with the open reconstruction of the distal femur articular surface with the help of KFI screws (Königsee, Allendorf, Germany). The 6.5cm long metaphyseal osseous defect of her femur was treated by shortening the femur with a T2-supracondylar nail (SCN) osteosynthesis (Stryker, Duisburg, Germany; Figure 2).

During further progression the osseous consolidation was delayed. For that reason, an autogenous cancellous iliac crest bone graft was carried out 3 months after the implantation of the SCN. Due to still missing consolidation after 9 further weeks, a second autologous cancellous and cortical bone graft out of her back iliac crest was implemented. During both surgeries the SCN was not revised. It followed a continuous progress of the osseous consolidation.

The leg shortening on the patient’s right side was primarily treated with a 6.5cm shoe sole compensation that did not satisfy the patient due to limited function and cosmetic reasons. Therefore a lengthening of the right femur was planned. With the help of a computed tomography scan the precise difference in leg length was measured and the eventual deviation in axis and rotation were defined. With these data the clinical-defined leg length difference of 6.5cm was confirmed; a deviation of the axis or torsion was ruled out. So 14 months after the trauma an ISKD was implanted.

**Figure 3:**
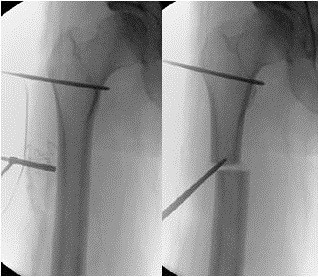
Drill-hole osteotomy.

**Figure 4:**
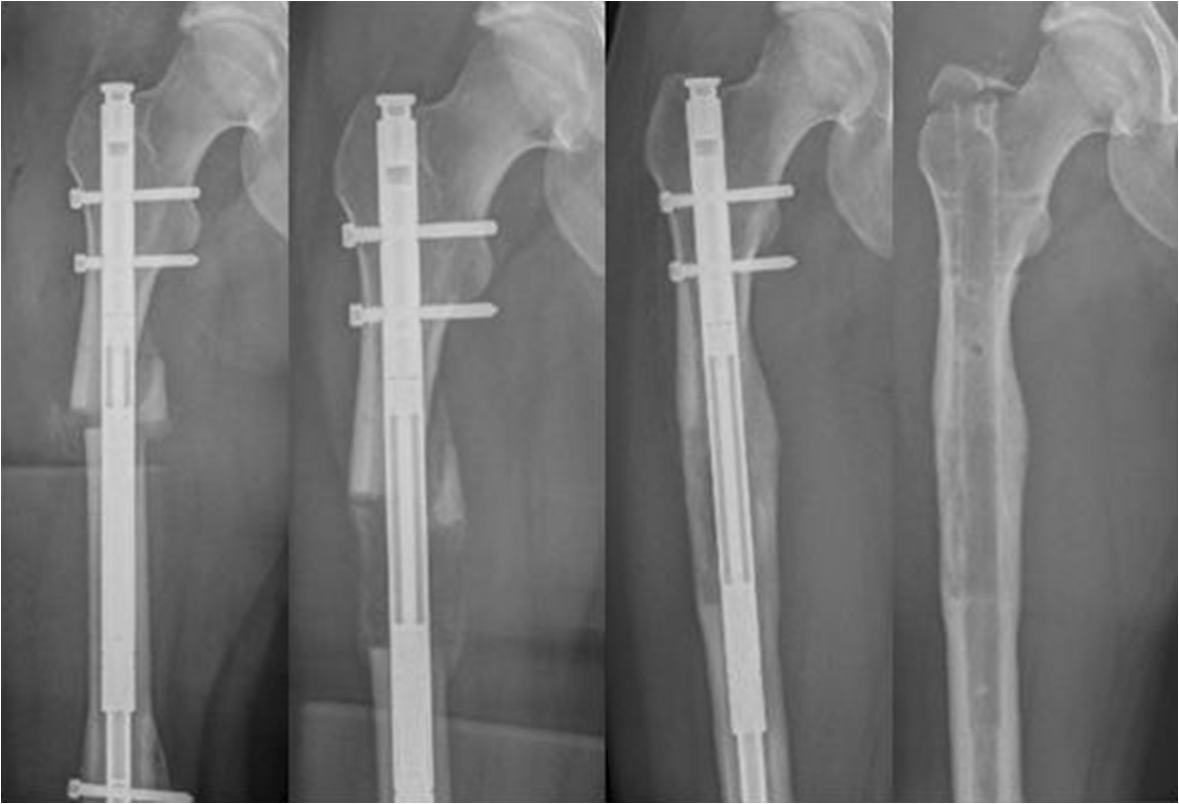
Process of osseous consolidation.

**Figure 5:**
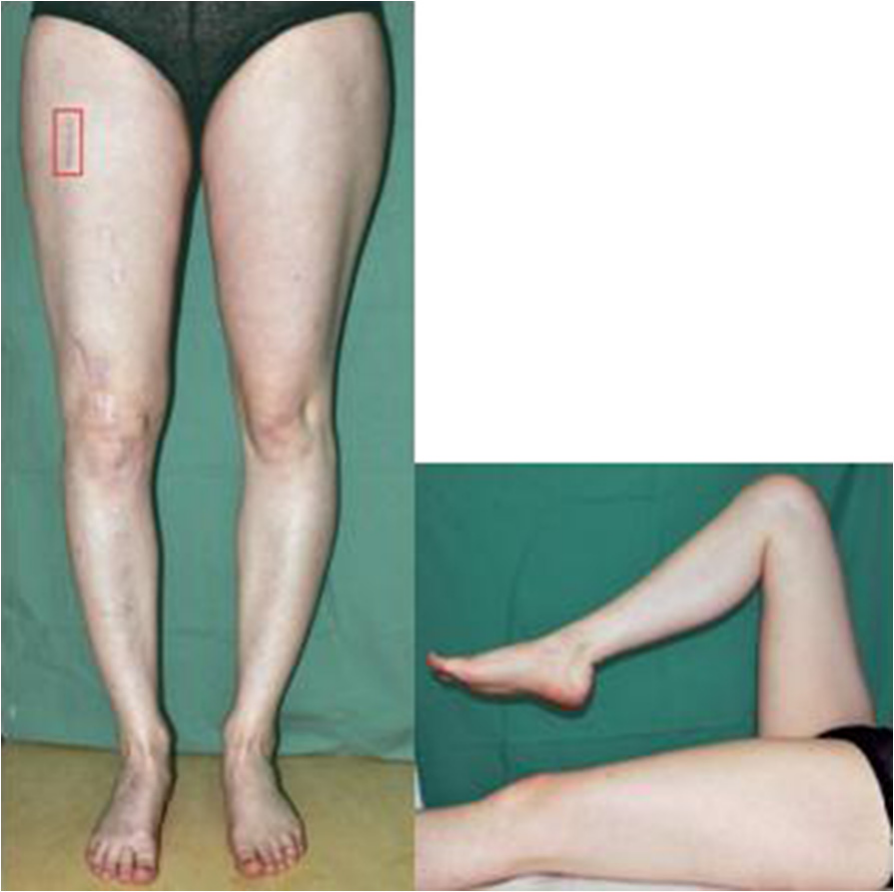
Complete compensation of leg length and good function of her right knee.

**Figure 6:**
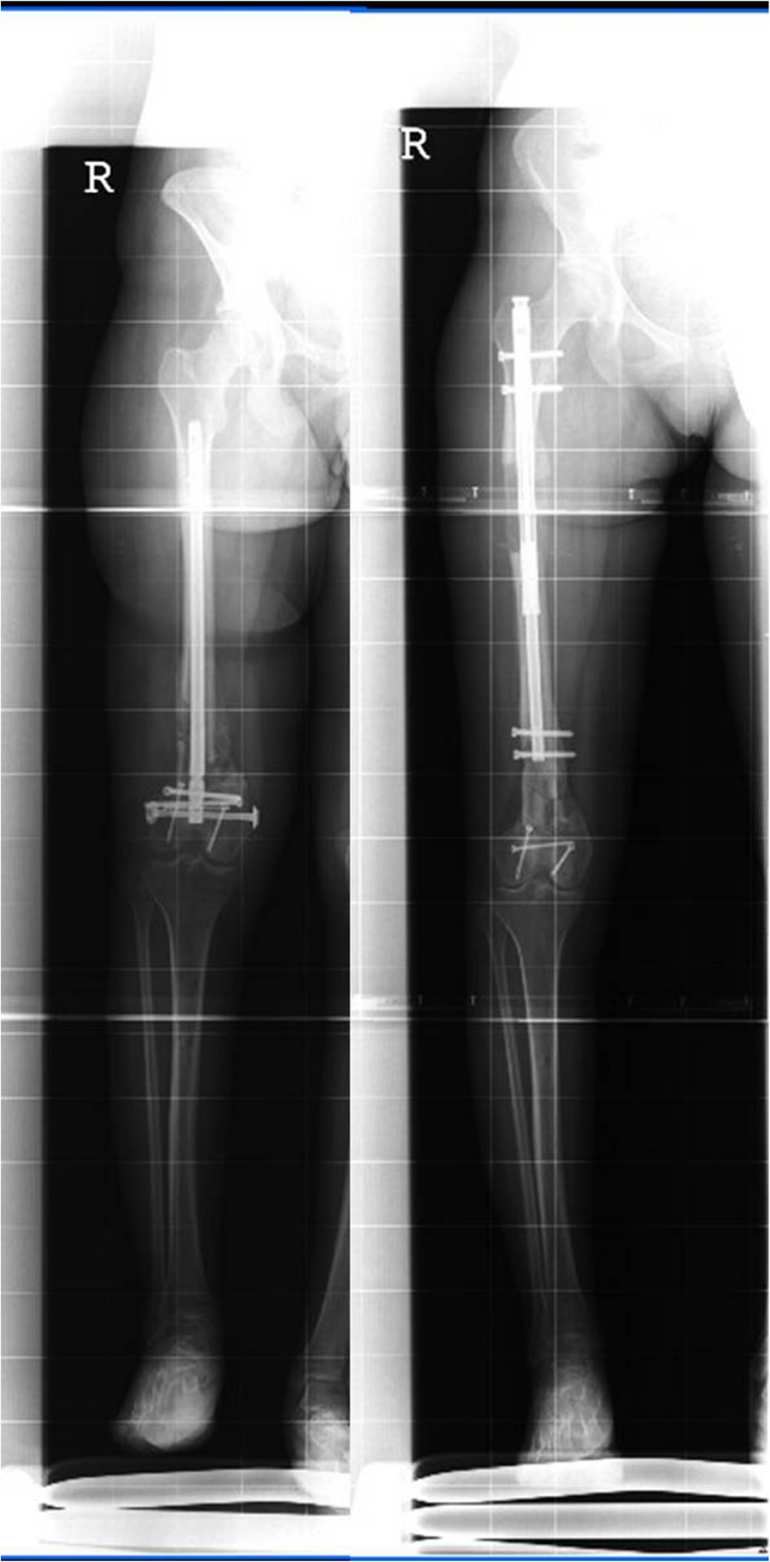
Full leg X-rays before and after distraction.

The procedure started with the removal of the SCN of the femur and followed with implantation of the ISKD in one sitting. In order to keep the bone necrosis and the soft tissue damage to a minimum a drill-hole osteotomy (Figure 3) was used. After that the intramedullary canal was prepared with a flexible reamer up to 14mm to gain an internal bone graft. An ISKD F 12.5mm caliber, 255 to 335mm nail was chosen. On the beginning of the fifth day, a distraction rate of 1mm a day was started. The patient was trained to handle the control monitor as well as the independent distraction mechanism. After releasing her into out-patient care, a sonographic or radiologic control was carried out on a weekly basis until the goal of 6.5cm lengthening was reached (Figure 4). In addition, an intensive physical therapy was implemented. Until reaching the lengthening maximum, the patient was mobilized through the use of a forearm crutch in order to relieve her right lower extremity. She temporarily had stretching pain on the iliotibial tract the more the distraction increased. Also there was a limited stretch function in her right knee. Both the pain and the limited function regressed quickly after the distraction procedure. Both complications were treated with physical therapy and nonsteroidal anti-inflammatory drugs. The entire length range was reached after 10 weeks as expected. Until the radiological consolidation of the distraction range, the patient was trained with partial weight-bearing of 20kg bodyweight. A radiological examination showed that the entire distraction range was filled with osseous material 9 months after the implantation (Figure 5). The ISKD was removed 2 years after implantation as specified by the manufacturer. Before the removal of the lengthening nail, the patient had no limitations in her daily life. Solely fast running and carrying heavy things led to pain in her right knee (Figure 6). The removal of the implant changed neither the clinical nor her subjective wellbeing.

## Discussion

Posttraumatic discrepancy of leg length leads to an awry pelvis and the compensation mechanism causes a scoliosis. This type of pathologic posture can cause severe pain in joints of the lower extremities and the spine. One method to compensate for the difference in length is orthopedic shoes. This results not only in a cosmetic problem for the patient, but also creates high costs. It can furthermore create a conflict with the patient’s profession and restriction of recreation mobility.

The use of an external fixation is the most common technique because of its capability of leg lengthening and correction in axis deformities [1,2]. This technique can be combined with an intramedular guidance to avoid axial deviation. The drawbacks of this universal technique are a series of complications such as pin infections, osteomyelitis, pin loosening, loss of surgical correction, refracture, and pain due to keloid and to the fixation of tissue that can occur. Not to mention an obviously limited amount of comfort, as well as psychological pressure on the patient due to the visibility of the external fixation [1-3].

By using an ISKD, complications that occur while using an external fixation can be avoided. Due to the intermedullary stabilization and leaving the implant in its place after distraction, secondary refractures and dislocation are avoided and weight-bearing rehabilitation is sped up. In 1978 such a full intramedullary distraction system was presented for the first time [4]. In addition to the ISKD, the Albizzia intramedullary nail is a further developed mechanic system. In order to activate the distraction mechanism of the Albizzia intramedullary nail, it is necessary to create a rotation movement of more than 20 degrees. This can cause severe pain which can often only be managed with help of a short anesthesia or even result in a discontinuation of the treatment [5,6]. A remaining rotational deformity is a specific complication of this system [7].

In this case report the limb length compensation was performed with, after ruling out any angular or rotational deformities, the implant of an ISKD. The ISKD nail described by Cole *et al*. has clearly less complications than the Albizzia nail [8]. Normal daily movement should create enough displacement of the ISKD in order to lengthen it properly.

To prevent common pitfalls like internal malfunction of the lengthening mechanism, runaway nails with insufficient bone regeneration or premature consolidation [2,8,9], the patient was evaluated carefully and started physical therapy months before the lengthening. The size of the implant and the site of the osteotomy were planned carefully. The implant had been checked thoroughly before implantation. As an intraoperative complication a fracture at the proximal osteotomy site was seen. We feel this was due to the wide diameter of the chosen nail and the muscular pull that also resulted in a slight abduction of the proximal fragment. Although we did not see any adverse effect this problem should be kept in mind when using an internal distraction system. Immediately after surgery a pain management system was established in collaboration with our pain therapists. During her in-patient stay, she was trained to use the control monitor. She was not released into out-patient care until she was able to perform the leg lengthening device on her own. After releasing her into out-patient care, a sonographic or radiological control followed on a weekly basis in order to control the lengthening process. After 10 weeks the whole lengthening distance was reached. The quick and safe osseous healing is, in our opinion, not least a result of minimal soft tissue damage during surgery, and an internal cancellous bone grafting.

## Conclusions

To balance out the limb length discrepancy of the femur after osteosynthesis of a difficult open distal femoral fracture, such as in this case, the ISKD is a suitable implant to use. In the case of the right indication, and by using it correctly, this device rises well above other lengthening devices being used currently.

## Consent

Written informed consent was obtained from the patient for publication of this case report and any accompanying images. A copy of the written consent is available for review by the Editor-in-Chief of this journal.

## Competing interests

The authors declare that they have no competing interests.

## Authors’ contributions

The work presented here was carried out in collaboration between all authors. All authors were involved in the patient’s treatment. SR, KK, and TM defined the scientific question. MD analyzed the data. KK, FG, and SR wrote the paper. TM and GOH discussed the report, interpretation and presentation. All authors read and approved the final manuscript.
